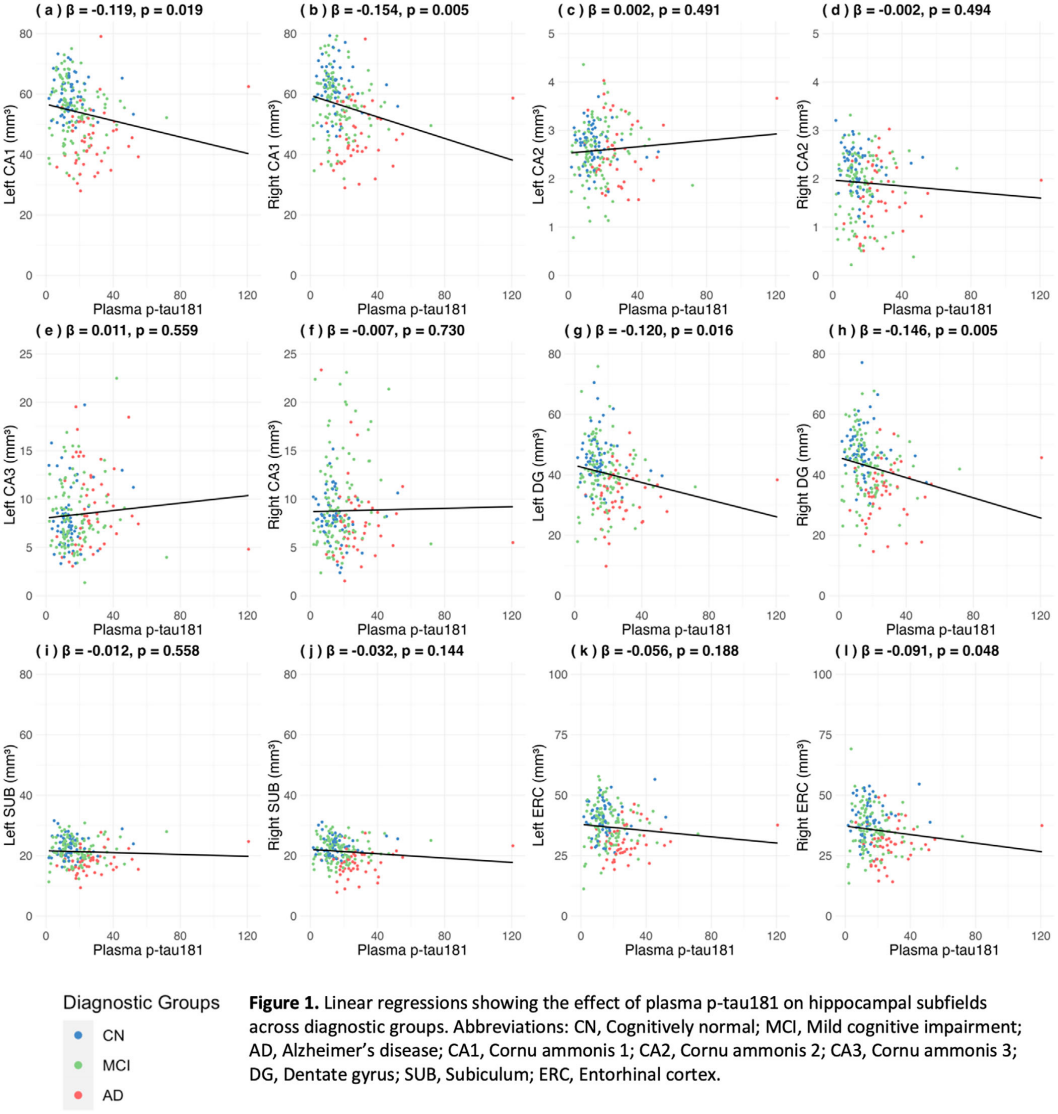# Differential associations between plasma p‐tau181 and hippocampal subfield integrity across the Alzheimer’s disease diagnostic continuum

**DOI:** 10.1002/alz.094608

**Published:** 2025-01-09

**Authors:** Adea Rich, Hwamee Oh

**Affiliations:** ^1^ Brown University, Providence, RI USA; ^2^ Alpert Medical School of Brown University, Providence, RI USA; ^3^ Carney Institute for Brain Science, Providence, RI USA

## Abstract

**Background:**

Plasma p‐tau181 is an increasingly established diagnostic marker for Alzheimer’s disease (AD); however, its precise relationship with brain tau pathology and the neural mechanisms underlying its association with cognitive impairment remain elusive. Our objective was to assess the association between plasma p‐tau181 and hippocampal (HC) subfield integrity and to investigate whether the subfields mediate the relationship between plasma p‐tau181 and cognition.

**Method:**

A total of 213 participants (57 cognitively normal, 109 mild cognitive impairment, and 47 AD) with plasma p‐tau181 measurements and high‐resolution T2‐weighted scans were selected from the Alzheimer’s Disease Neuroimaging Initiative (ADNI). HC subfield volume was measured at baseline using the Automatic Segmentation of Hippocampal Subfields (ASHS) software. Follow‐up HC subfield volume collected around one year was measured for eighty‐nine participants. A linear regression adjusted for age and sex evaluated the relationship between plasma p‐tau181 and HC subfields at baseline, as well as HC volume rate of change over one year. A mediation model assessed whether HC subfields mediate the association between plasma p‐tau181 and memory and executive functioning.

**Result:**

Our findings indicate that increasing levels of plasma p‐tau181(pg/mL) are associated with decreased volume in the left CA1 (β = ‐0.119, p = 0.019, **Fig. 1a**), right CA1 (β = ‐0.154, p = 0.005, **Fig. 1b),** left dentate gyrus (β = ‐0.120, p = 0.016, **Fig. 1g)**, right dentate gyrus (β = ‐0.146, p = 0.005, **Fig. 1h)**, and right entorhinal cortex (β = ‐0.091, p = 0.048, **Fig. 1l)**. Moreover, these subfields partially mediate the relationship between plasma p‐tau181 and memory and executive functioning composite scores. Baseline plasma p‐tau181 did not predict longitudinal atrophy of the HC subfields across diagnostic groups.

**Conclusion:**

Our study reveals consistent associations between plasma p‐tau181 levels and vulnerable HC subfields, including CA1, entorhinal cortex, and dentate gyrus, collectively implicated in normal aging and Alzheimer’s disease pathologies. Volumetric changes in CA1, dentate gyrus, and right entorhinal cortex were shown to underlie the association of plasma p‐tau181 with both memory and executive functioning. Together, our findings indicate that selective changes in HC subfield volume serve as a neural basis underlying the relationship between plasma p‐tau181 levels and cognitive impairment.